# Investigation of three enzymes and their roles in the embryonic development of parthenogenetic *Haemaphysalis longicornis*

**DOI:** 10.1186/s13071-020-3916-7

**Published:** 2020-01-31

**Authors:** Zhao-Xi Qiu, Yuan Li, Meng-Meng Li, Wen-Ying Wang, Tian-Tian Zhang, Jing-Ze Liu

**Affiliations:** Hebei Key Laboratory of Animal Physiology, Biochemistry and Molecular Biology, College of Life Sciences, Hebei Normal University, Shijiazhuang, 050024 China

**Keywords:** Parthenogenesis, *Haemaphysalis longicornis*, Embryogenesis, Cathepsin B, Cathepsin D, Acid phosphatase

## Abstract

**Background:**

The tick *Haemaphysalis longicornis* exhibits two separate reproductive populations: bisexual and parthenogenetic, which have diploid and triploid karyotypes, respectively. The parthenogenetic population can undergo engorgement without copulation and produce viable female-only offspring with a longer incubation period than the bisexual population. Three enzymes, cathepsin B, cathepsin D and acid phosphatase, were found to be involved in vitellin degradation during the embryonic development of bisexual *H. longicornis*. However, the expression and activity profiles of these enzymes during the embryonic development of parthenogenetic ticks remain unknown. In the present study, the transcriptional expression profile, enzyme activity and roles in embryogenesis of the three enzymes during the embryonic development of parthenogenetic *H. longicornis* were investigated.

**Methods:**

Quantitative real-time polymerase chain reaction (qPCR) and fluorescence detection were used to analyze the dynamic changes in the three enzymes during embryogenesis. The roles of the three enzymes during embryogenesis were also explored using RNA interference (RNAi).

**Results:**

The three enzymes were all expressed during embryonic development in parthenogenetic *H. longicornis*. The expression of *cathepsin B* was highest on day 15, whereas that of *cathepsin D* was highest on day 3 and the peak of *acid phosphatase* expression occurred on day 9. The activity of cathepsin B was highest on day 3 and lowest on day 5, then gradually increased and remained stable. Cathepsin D activity was highest on day 1 and showed a gradually decreasing trend, whereas acid phosphatase showed the opposite trend and reached a peak on day 23. RNA interference experiments in engorged female ticks revealed that there was no significant difference in the number of eggs laid, but the hatching rate of the eggs was significantly decreased.

**Conclusion:**

The three enzymes all play important roles in embryonic development of *H. longicornis*, but the expression patterns and changes in the activity of the enzymes in the bisexual and parthenogenetic populations are different. The results will help a better understanding of the similarities and differences underlying embryonic development in the bisexual and parthenogenetic populations and contribute to the future exploration of the development of the parthenogenetic population of *H. longicornis*.
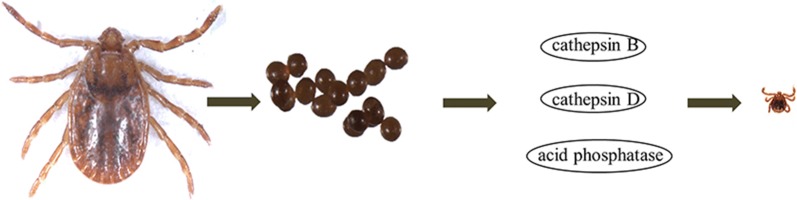

## Background

Ticks are obligate blood-sucking ectoparasites and over 900 tick species have been reported around the world [1]. Ticks are the world’s second most common vector of disease after mosquitoes, exhibiting a wide range of hosts, including mammals, birds, reptiles and amphibians [2]. They can transmit a variety of pathogens, including viruses, bacteria, rickettsiae, helminths and protozoans [3]. In recent years, ticks have been shown to cause severe fever with thrombocytopenia syndrome [4, 5], human granulocytic anaplasmosis [6] and African swine fever [7], which affect human health and cause significant losses to the livestock industry.

*Haemaphysalis longicornis* is a three-host tick that is widely distributed in Australia, New Zealand, Korea, Japan and China [8]. Recently, it was found in Hunterdon County, New Jersey, USA for the first time [9]. It is an essential vector of zoonotic agents and can transmit severe fever with thrombocytopenia syndrome virus and *Anaplasma*, *Babesia*, *Borrelia*, *Ehrlichia* and *Rickettsia* bacteria [10]. *Haemaphysalis longicornis* exhibits two reproductive populations: bisexual and obligate parthenogenetic populations [11]. Comparison of the morphological characteristics of the two populations revealed that parthenogenetic individuals (except for engorged females) are significantly greater in weight than bisexual individuals, the genital apron of parthenogenetic females is wider than that of bisexual females and the Haller’s organ of parthenogenetic individuals has no seam hole structure [12, 13]. In addition, bisexual and parthenogenetic *H. longicornis* are diploid and triploid, respectively. Parthenogenetic *H. longicornis* can complete their life-cycle without fertilization, without any males being observed throughout the life-cycle and they show strict reproductive isolation from the bisexual population [11, 12]. Because of this unique mode of reproduction, the feeding period of parthenogenetic females is significantly shorter than that of bisexual females, possibly due to the mating behavior of the bisexual population. However, parthenogenetic individuals show a slightly longer development cycle than bisexual individuals, including nymphal premolting, preoviposition, oviposition and egg incubation periods. Although the egg weight of the parthenogenetic population is greater than that of the bisexual population, the hatching rate is lower [11, 12].

Embryonic development is an important physiological process in reproduction and development. Many proteolytic enzymes accumulate in oocytes together with vitellin and regulate vitellin degradation to provide nutrients during embryonic development [14]. This physiological process has been observed in many insects, such as *Bombyx mori* [15–17], *Rhodnius prolixus* [18, 19], *Periplaneta americana* [20], *Blattella germanica* [21], *Culex quinquefasciatus* [22], *Musca domestica* [23], *Anticarsia gemmatalis* [24], *Dipetalogaster maxima* [25], *Spodoptera exigua* [26] and *Helicoverpa armigera* [27]. However, related studies have only been reported in several tick species, including *Ornithodoros moubata* [28], *Rhipicephalus microplus* [14, 29–33] and bisexual *H. longicornis* [34]. Our previous study has proven that three enzymes, cathepsin B, cathepsin D and acid phosphatase, are involved in vitellin degradation during the embryonic development of bisexual *H. longicornis* [34]. However, the expression and activity profiles of these enzymes during the embryonic development of parthenogenetic ticks remain unknown. In the present study, the transcriptional expression profiles, enzyme activity and roles of the three enzymes during the embryonic development of parthenogenetic *H. longicornis* were investigated. The results will contribute to a better understanding of the similarities and differences underlying embryonic development between the bisexual and parthenogenetic populations and to the future exploration of the development of the parthenogenetic population of *H. longicornis*.

## Methods

### Tick collection and rearing

Parthenogenetic *H. longicornis* ticks were collected by flag dragging from Cangxi County (31°37ʹ–32°10″N, 105°43ʹ–106°28″E) in Sichuan Province, China. They were maintained for several generations in our laboratory and it was verified that no males were observed throughout the entire life-cycle to ensure that the ticks were obligate parthenogenetic. These ticks were reared on the ears of rabbits in the laboratory as described previously [35]. After finishing a blood meal, the engorged ticks were placed in clean, plastic Petri plates for oviposition. The plates were maintained under standard environmental chamber conditions (26 ± 1 °C, 75 ± 5% RH and a 8:16 h L:D photoperiod). The eggs were collected and separated after 1, 3, 5, 7, 9, 11, 13, 15, 17, 19, 21 and 23 days and snap frozen using liquid nitrogen before being stored at − 80 °C for further use. All eggs used in this research originated from female ticks of the same batch.

### Primer design and sequence analysis

Primers for quantitative analysis were designed using sequence information from the National Center for Biotechnology Information (NCBI). Because of the paucity of sequences from parthenogenetic *H. longicornis*, the sequences of several ticks were aligned and compared to search for conservative sequences upon which to design corresponding primers. For *cathepsin B*, sequences from *H. longicornis* (GenBank: AB255051), *Ixodes ricinus* (GenBank: EF428206) and *Ixodes scapularis* (GenBank: XM_002435418) were used. For *cathepsin D*, sequences from *H. longicornis* (GenBank: EU019715), *I. ricinus* (GenBank: HQ615697) and *R. microplus* (GenBank: FJ655904) were used. For *acid phosphatase*, sequences from *H. longicornis* (GenBank: HM150759), *I. scapularis* (GenBank: XM_002410276) and *Anoplophora glabripennis* (GenBank: XM_018710438) were used. The primers for the *β-actin* gene were designed using the sequence from *H. longicornis* (GenBank: AY254898). All primers were designed with Primer Premier 5 (Premier Biosoft) and had lengths of 18–22 bp and were free of dimers and hairpins (Table 1). PCR amplification was performed in a total volume of 10 μl, which included 5 μl of 2× Taq PCR StarMix (GenStar BioSolutions), 4 μl of H_2_O, 0.6 μl of cDNA template and 0.2 μl of each 10 μM primer (Table 1). The PCR conditions were as follows: initial denaturation at 94 °C for 3 min, followed by 35 cycles of 94 °C for 30 s, 60 °C for 30 s and 72 °C for 30 s, with a final extension step at 72 °C for 10 min. The PCR assays were performed in a ProFlex™ 3 × 32-well PCR System (Applied Biosystems). The PCR products were checked on a 1% agarose gel and positive amplicons were purified using an AxyPrep^TM^ DNA Gel Extraction Kit (Axygen). The purified products were inserted into the pEASY^®^-T1 Simple Cloning Vector (TransGen), which was then transformed into *Escherichia coli* DH5α cells (Invitrogen). The transformed cells were submitted to Sangon (Sangon Biotech) and the resulting sequences were analyzed by BLASTn searches in the NCBI database to ensure that the primers were correct.

**Table 1:** Sequences of different primer sets used for qPCR

Gene	Primer sequence (5ʹ-3ʹ)	Amplicon size (bp)
*Cathepsin B*	F: GCGTGGAGCTACTGGGTG	133
	R: TGCTCTTGTCGCAGGGTC	
*Cathepsin D*	F: CGGCGTGAAAGTAGGCGATAA	88
	R: CGGCCCAGCAATCAAGGAG	
*Acid phosphatase*	F: CACGCACAAAGGTAAAAA	180
	R: CACACTTTCTTGTCCCGT	
*β-actin*	F: CGTTCCTGGGTATGGAATCG	70
	R: TCCACGTCGCACTTCATGAT	

### qPCR analysis

Total RNA was extracted from eggs in different developmental stages (80 mg each) using an AxyPrep™ Multisource Total RNA Miniprep Kit (Axygen). Then, total RNA from each sample was reverse transcribed to cDNA using TransScript® One-Step gDNA Removal and cDNA Synthesis SuperMix (TransGen Biotech) following the manufacturer’s protocol. qPCR was performed in an Mx3005P system (Agilent Technologies) using TransStart® Top Green qPCR SuperMix (TransGen Biotech) following the manufacturer’s instructions. The reaction system was as follows: 10 μl of 2× TransStart® Top Green qPCR SuperMix, 7.8 μl of H_2_O, 1 μl of cDNA template, 0.4 μl of Passive Reference Dye (TransGen Biotech) and 0.4 μl of each primer at 10 μM (Table 1). The parameters of the machine were set as described previously [34]. The results were normalized to *β-actin* and analyses of gene expression were performed using the 2^−ΔΔCq^ method [36].

### Enzyme activity assays

Total protein was extracted from eggs at different developmental stages (1.0 g each) by using 50 mM sodium acetate buffer (pH 5.0). Then, the homogenates were centrifuged at 12,000 × *rpm* for 10 min at 4 °C to obtain the supernatants. The bicinchoninic acid (BCA) method was used to adjust the protein concentration for consistency [37]. Cathepsin B and Cathepsin D Activity Fluorometric Assay Kits (Biovision, Milpitas, USA) were used to identify the activity of the enzymes following the manufacturer’s instructions. Cathepsin B can cleave the synthetic substrate RR-amino-4-trifluoromethyl coumarin (RR-AFC) to release free AFC, which can be easily quantified using a fluorometer or fluorescence plate reader at Ex/Em = 400/505 nm. Cathepsin D acts on the synthetic substrate GKPILFFRLK(Dnp)-D-R-NH2-MCA to release fluorescence, which can be easily quantified using a fluorometer or fluorescence plate reader at Ex/Em = 328/460 nm. An Acid Phosphatase Activity Colorimetric Assay Kit (Biovision) was used to identify the activity of acid phosphatase. The phosphatase substrate p-nitrophenyl phosphate (pNPP) was dephosphorylated by acid phosphatase so that it turned yellow and could then be detected at λmax of 405 nm.

### RNAi

Primers for RNAi were designed in the same way as the qPCR primers. The TAA TAC GAC TCA CTA TAG G (T7) promoter sequence was added to the 5ʹ end of the primers (Table 2). Primer sequences for the control gene green fluorescent protein (*GFP*) from *Tetraselmis subcordiformis* (GenBank: KJ668651) were also used. The PCR products were gel purified to synthesize RNA by using the T7 RiboMAX^TM^ Express RNAi System (Promega, Madison, USA) according to the manufacturer’s protocol. Double-stranded RNA (dsRNA) injection was performed as described previously [38]. Engorged female ticks were microinjected with 4 μg of the enzyme-targeted dsRNAs in a volume of 1 μl. Thirty ticks were used for each group. The engorged ticks were placed in clean, plastic Petri plates for oviposition. The plates were maintained under standard environmental chamber conditions (26 ± 1 °C, 75 ± 5% RH and a 8:16 h L:D photoperiod). The eggs were collected daily until the ticks no longer laid eggs and the number of eggs in each group was counted. In the cathepsin B group, eggs were collected at 11, 13, 15 and 17 days to confirm gene-specific silencing by qPCR [39]. A portion of the eggs were used to calculate the hatching rate. The same procedure was followed for the cathepsin D (3, 5, 7 and 9 days), acid phosphatase (9, 11, 13 and 15 days) and control (3, 5, 7, 9, 11, 13, 15 and 17 days) groups.

**Table 2:** Sequences of different primer sets used for RNAi

Gene	Primer sequence (5ʹ-3ʹ)	Amplicon size (bp)
*Cathepsin B* (T7)	F: TAATACGACTCACTATAGGATTGTCCACCTCGCTGCC	430
	R: TAATACGACTCACTATAGGGGTCCGTGTGCCTCTGGT	
*Cathepsin D* (T7)	F: TAATACGACTCACTATAGGTGTTCGACACCGGCTCCT	497
	R: TAATACGACTCACTATAGGCTGCCAGTAGCCCTTGCG	
*Acid phosphatase* (T7)	F: TAATACGACTCACTATAGGGGTCACATCACGCACAAA	539
	R: TAATACGACTCACTATAGGTGCAGGGTGCTGTTGTAG	
*GFP* (T7)	F: TAATACGACTCACTATAGGGACGTAAACGGCCACAAGT	583
	R: TAATACGACTCACTATAGGGCTTCTCGTTGGGGTCTTT	

### Statistical analysis

All experiments were repeated three times and the data were analyzed using ANOVA with SPSS 12.0 software (IBM). The t-test statistical analysis was performed using the SAS JMP statistical program 13.2 (SAS Institute Inc). The differences were considered statistically significant when *P* ≤ 0.05. Experimental values were obtained from three independent assays and expressed as the mean ± standard error.

## Results

### Cloning of the *cathepsin B*, *cathepsin D* and *acid phosphatase* genes for qPCR

**Fig. 1:**
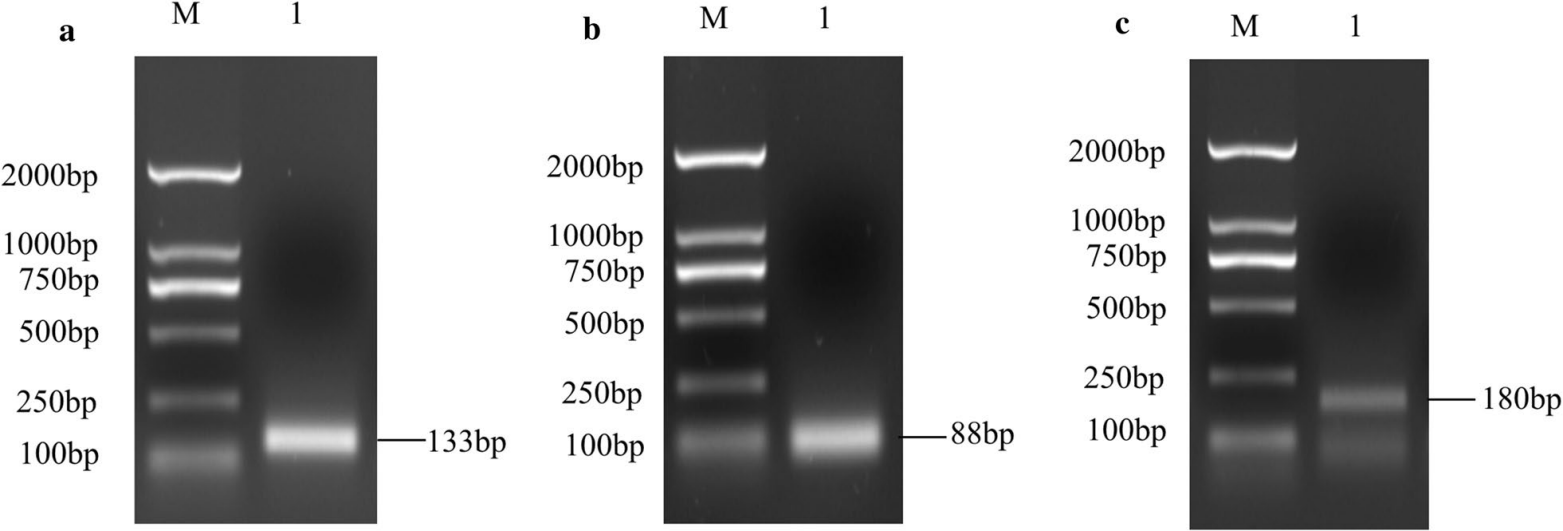
qPCR amplification products analysis of *cathepsin B* (**a**), *cathepsin D* **(b**) and *acid phosphatase* (**c**) of the parthenogenetic *H. longicornis*

Total RNA was extracted from eggs collected at 1, 3, 5, 7, 9, 11, 13, 15, 17, 19, 21 and 23 days and then converted into cDNA. A portion of these three genes were amplified and the PCR results showed that the sizes of amplicons for the *cathepsin B*, *cathepsin D* and *acid phosphatase* genes were 133 bp, 88 bp and 180 bp, respectively (Fig. 1a–c). The deduced amino acid sequences of the three enzymes showed 100% similarity with those of bisexual *H. longicornis*.

### Expression and activity of *cathepsin B* during embryogenesis

**Fig. 2:**
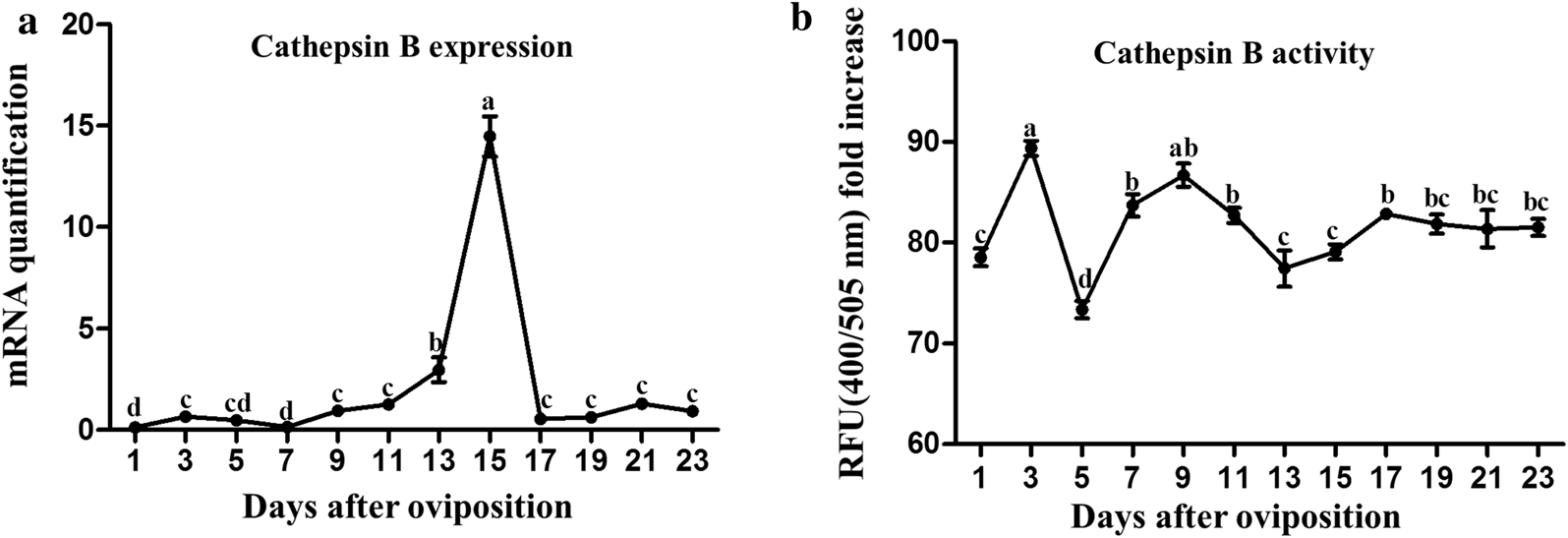
Dynamic changes of cathepsin B during embryonic development of the parthenogenetic *H. longicornis*. **a** Gene transcripts of *cathepsin B*. **b** Activity of cathepsin B. The levels of gene mRNA expression were normalised against the mRNA of *β-actin* and the error bars represent the mean ± SE values and the letters (a, b, c, d) labels represent significant differences (*P* ≤ 0.0001)

To illustrate the functions of cathepsin B during embryogenesis, the transcriptional expression profile of *cathepsin B* was monitored *via* qPCR. The results indicated that *cathepsin B* was expressed throughout embryonic development (Fig. 2a). The expression of *cathepsin B* was low before day 11, then rapidly rose to its highest level on day 15 (*F*_(11, 24)_ = 129.77, *P* ≤ 0.0001). However, the expression of *cathepsin B* decreased to a low level after day 15.

The analysis in which the fluorescent substrate was used to detect the activity of cathepsin B showed that its activity was high throughout embryonic development (Fig. 2b). On day 3, its activity reached the highest level (*F*_(11, 24)_ = 14.98, *P* ≤ 0.0001), but it decreased to the lowest level on day 5 and increased to a stable level thereafter.

### Expression and activity of *cathepsin D* during embryogenesis

**Fig. 3:**
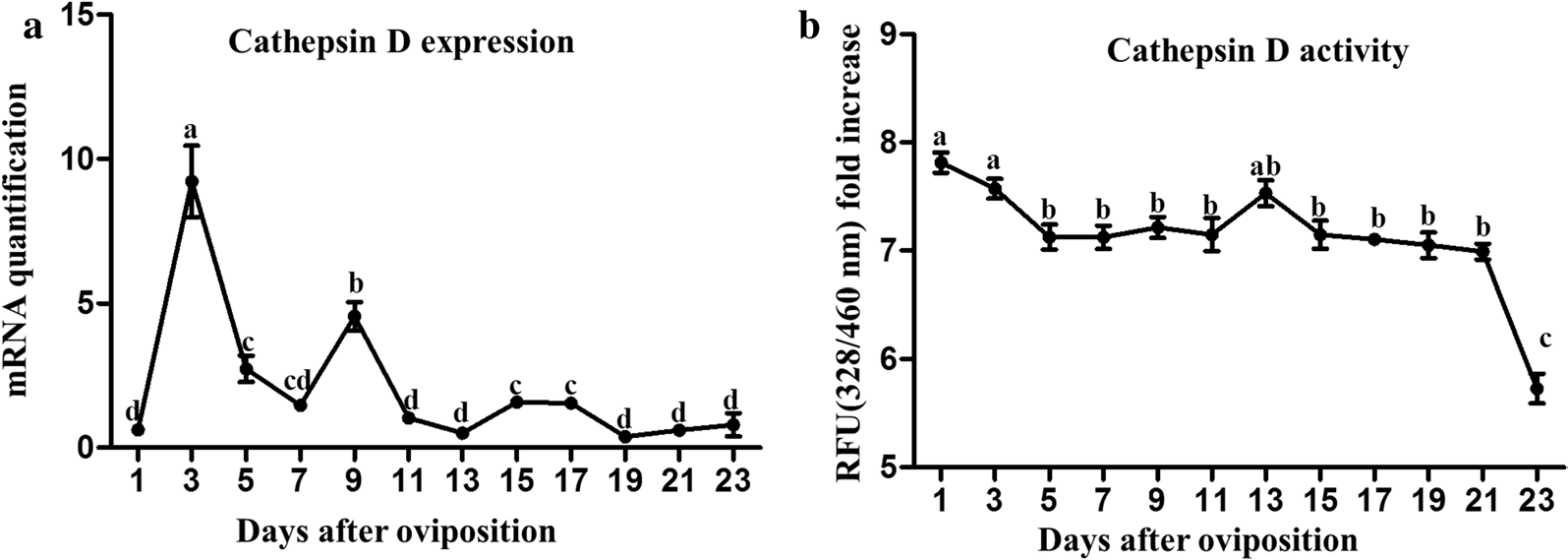
Dynamic changes of cathepsin D during embryonic development of parthenogenetic *H. longicornis*. **a** Gene transcripts of *cathepsin D*. **b** Activity of cathepsin D. The levels of gene mRNA expression were normalised against the mRNA of *β-actin* and the error bars represent the mean ± SE values and the letters (a, b, c, d) labels represent significant differences (*P* ≤ 0.0001)

The results showed that the expression of *cathepsin D* was detectable in the eggs from different stages of embryonic development (Fig. 3a). The transcriptional level was very low on the first day but increased rapidly to the highest level on day 3 (*F*_(11, 24)_ = 33.59, *P* ≤ 0.0001). Thereafter, the expression level gradually declined. The activity of cathepsin D was highest on day 1 (*F*_(11, 24)_ = 21.15, *P* ≤ 0.0001) and decreased on days 3 and 5 (Fig. 3b). Thereafter, its activity was relatively stable until day 21, but it fell sharply on day 23.

### Expression and activity of acid phosphatase during embryogenesis

**Fig. 4:**
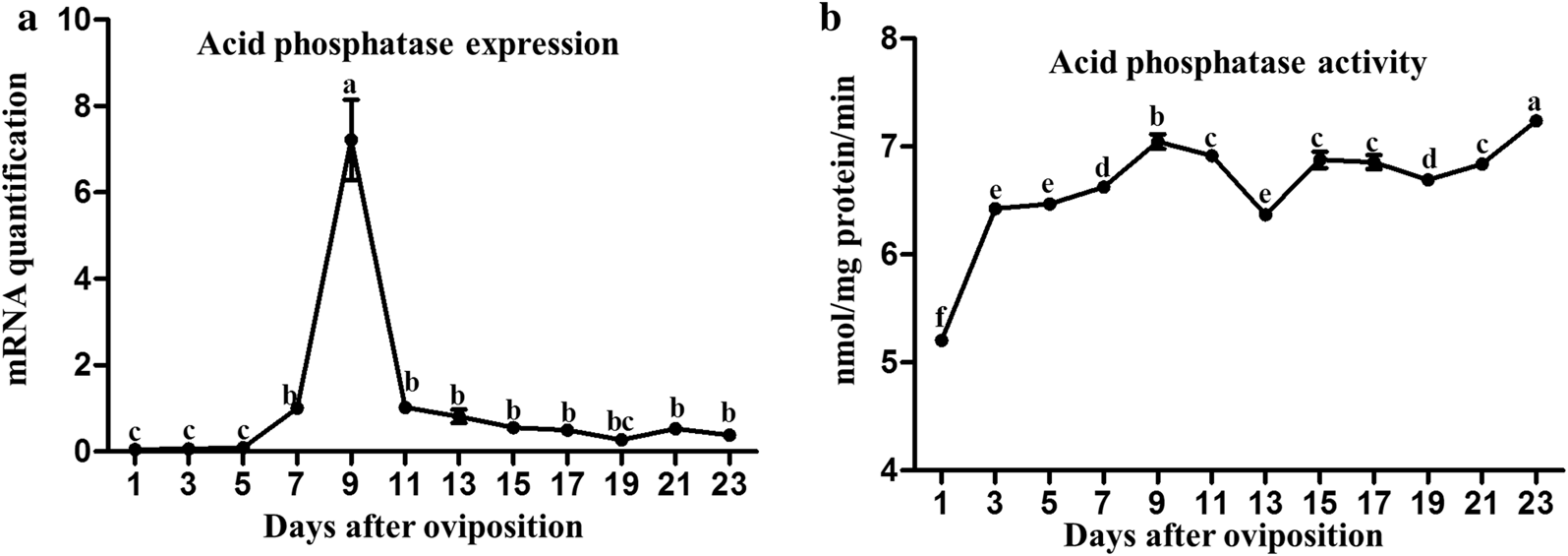
Dynamic changes of acid phosphatase during embryonic development of parthenogenetic *H. longicornis*. **a** Genes transcripts of *acid phosphatase*. **b** Activity of acid phosphatase. The levels of gene mRNA expression were normalised against the mRNA of *β-actin* and the error bars represent the mean ± SE values and the letters (a, b, c, d, e, f) labels represent significant differences (*P* ≤ 0.0001)

On the first five days, the *acid phosphatase* gene showed very little expression (Fig. 4a). After day 5, its expression increased and was highest on day 9 (*F*_(11, 24)_ = 50.14, *P* ≤ 0.0001). Thereafter, its expression dropped to a low level, where it remained until the end of embryogenesis. The activity trend of acid phosphatase showed a gradual increase until reaching the highest level on day 23 (Fig. 4b, *F*_(11, 24)_ = 145.08, *P* ≤ 0.0001).

### Gene cloning and assessment of gene silencing

**Fig. 5:**
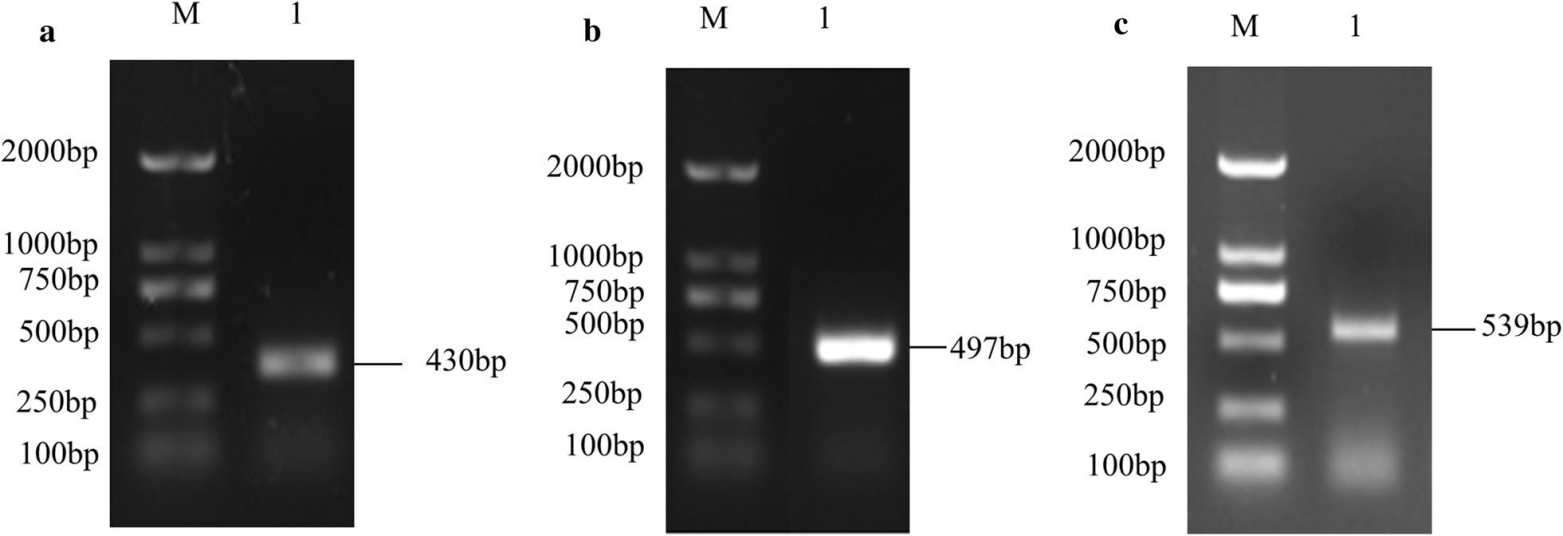
RNAi PCR amplification products analysis of *cathepsin B* (**a**), *cathepsin D* (**b**) and *acid phosphatase* (**c**) of parthenogenetic *H. longicornis*

**Fig. 6:**
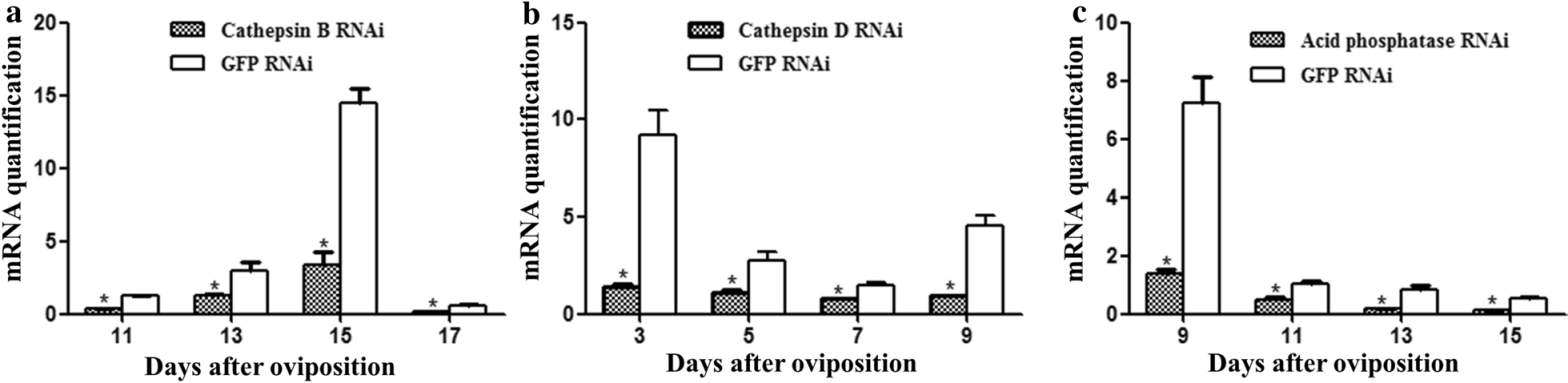
Assessment of genes silencing efficiency of *cathepsin B* (**a**), *cathepsin D* (**b**) and *acid phosphatase* (**c**) of parthenogenetic *H. longicornis*. The levels of gene mRNA expression were normalised against the mRNA of *β-actin* and the error bars represent the mean ± SE values; asterisks indicate represent significant differences (*P* ≤ 0.05)

A portion of these three genes were amplified and the amplified fragment sizes of *cathepsin B*, *cathepsin D* and *acid phosphatase* were 430 bp, 497 bp and 539 bp, respectively (Fig. 5a–c). After microinjection, we collected the eggs of the experimental and control groups at different developmental stages to detect the expression of the enzyme-encoding genes by qPCR. The dsRNA-mediated knockdown of the transcripts of *cathepsin B* (*F*_(3, 11)_ = 11.13, *P* = 0.0032), *cathepsin D* (*F*_(3, 11)_ = 7.57, *P* = 0.0101) and *acid phosphatase* (*F*_(3, 11)_ = 126.43, *P* ≤ 0.0001)resulted in a decrease in their relative levels compared to *GFP* (Fig. 6a–c). These results verified that the RNAi knockdown of enzyme-encoding genes for engorged female ticks was effective.

### Effect of RNAi targeting enzyme-encoding genes on oviposition and hatching rates

**Fig. 7:**
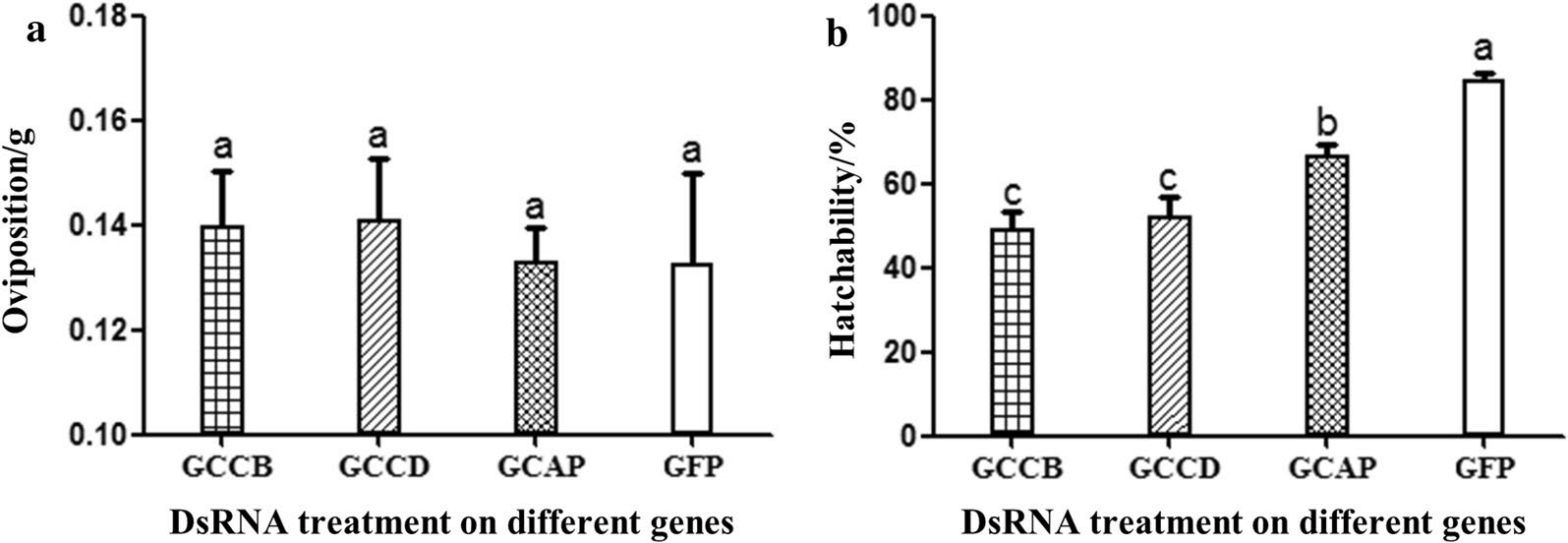
DsRNA-mediated knockdown of transcripts for *cathepsin B* (CB), *cathepsin D* (CD) and *acid phosphatase* (AP) affect oviposition (**a**) and eggs’ hatchability (**b**) of parthenogenetic *H. longicornis*. The error bars represent the mean ± SE values and the letter (a, b, c) labels represent significant differences (*P* ≤ 0.0001)

The engorged ticks of the experimental and control groups were maintained in the incubator to lay eggs. The oviposition of the ticks and the hatching rate of the eggs were recorded. The weights of the eggs laid by the ticks in the *cathepsin B*, *cathepsin D*, *acid phosphatase* and *GFP* groups were 140 mg, 140 mg, 130 mg and 130 mg, respectively (Fig. 7a). There was no significant difference between the groups (*F*_(3, 8)_ = 0.129, *P* = 0.940). However, the hatching rates of these groups were 49.33%, 52.17%, 66.40% and 84.87%, respectively (Fig. 7b). A significant difference was observed between the experimental and control groups (*F*_(3, 8)_ = 22.127, *P* ≤ 0.0001).

## Discussion

The main biochemical event in the embryonic development of arthropods is the utilization of yolk components [31]. As the major yolk protein, vitellin is the largest source of energy and nutrients during embryonic development and plays an irreplaceable role. Vitellin is still present in the larvae and is degraded in an orderly manner under the regulation of hydrolases [40]. The hydrolases stored in structures such as the modified lysosomes referred to as yolk granules are inactive proenzymes and they are activated mainly by developmentally controlled acidification [41]. These hydrolases can be classified into four categories: cysteine proteases (cathepsin B), serine proteases, aspartic proteases (cathepsin D) and acid phosphatases.

Cathepsin B is a cysteine protease found in the cytolysosomes that plays a vital role in the degradation of yolk during embryogenesis. In our study, the expression of *cathepsin B* was low on days 1–11 and rapidly increased to reach the highest level on day 15, then decreased sharply in the late development stage. However, in bisexual *H. longicornis*, *cathepsin B* showed a rapid rising trend on days 1–5, followed by a slow decline on later days [34]. The expression differences between the two populations might be due to the longer incubation of parthenogenetic eggs [12]. In other species, *cathepsin B* presents a high expression level at different developmental stages [42, 43]; therefore, in parthenogenetic *H. longicornis*, *cathepsin B* is more likely to be highly expressed in late embryonic development. The activity of cathepsin B fluctuated and was maintained at a high level throughout embryonic development in the parthenogenetic population, but in the bisexual population, it gradually decreased. Combined with the results obtained for cathepsin B expression, it can be concluded that cathepsin B played a vital role in late embryonic development in the parthenogenetic population, while in the bisexual population, cathepsin B tended to play a role in the early stage.

Cathepsin D is the major lysosomal aspartic protease and is widely distributed in the cells, where it regulates programmed cell death, autophagy and the degradation of yolk protein [18, 25]. Our study showed that the expression of *cathepsin D* rapidly reached the highest level on day 3 and then gradually decreased to a low level in the later development stage. The activity of cathepsin D was also highest in the early stage (on day 1) and was maintained at a high level during the later stage, except on day 23. However, in bisexual *H. longicornis*, the expression and activity of cathepsin D were highest on days 11 and 13, respectively [34]. In contrast to cathepsin B, in parthenogenetic *H. longicornis*, cathepsin D tended to play a role in the early stage, while in the bisexual population, cathepsin D was more likely to regulate embryogenesis in the late stage. Although both cathepsin B and D were indicated to regulate embryogenesis, their dynamic changes were not the same in the parthenogenetic and bisexual populations. In our previous work, we found that the vitellin of the parthenogenetic population exhibits 9 subunits, ranging from 31 kDa to 203 kDa; however, in the bisexual population, vitellin exhibits only 8 subunits, ranging from 52 kDa to 112 kDa [44, 45]. This might be another reason for the different dynamic changes in the enzymes, in addition to the longer incubation period.

In contrast to cathepsins B and cathepsins D, acid phosphatase is a classical lysosomal enzyme that catalyzes the hydrolysis of orthophosphoric monoesters [24]. The expression of the enzyme in the parthenogenetic and bisexual populations was highest on days 9 and 11, respectively. Its activity rose in the early stage (1–5 days) and was maintained at a high level on the following days in both populations [34]. The dynamic changes in acid phosphatase in the two populations were similar, possibly because the presence of acid phosphatase facilitates the degradation of yolk protein by the other two enzymes [18–20, 23, 25].

After RNAi treatment, the expression of the three targeted genes in the eggs was found to be significantly suppressed and the hatching rate was significantly reduced compared with that in the GFP group. However, the oviposition of the ticks showed no significant change. Similar results were previously found in bisexual *H. longicornis* [46, 47]. In *Radopholus similis* [48], *Schistosoma mansoni* [49, 50], *Schistosoma japonicum* [51] and *Schmidtea mediterranea* [52], knocking down the *cathepsin B* gene affects the development of the eggs and the hatching rate and even slows development and results in a shorter body length. In *B. mori*, cathepsin B participates in programmed cell death during metamorphosis and RNAi knockdown of *cathepsin B* leads to the stagnation of the larval-pupal metamorphosis [53]. The role of cathepsin D in the metamorphosis of *S. exigua* has also been reported and RNAi knockdown of *cathepsin D* reduces the survival rate in the fifth-instar [26]. A similar result was found in *B. mori*, in which deficiency of cathepsin D affects the pupation of larvae [54]. RNAi knockdown of *cathepsin D* in *S. mansoni* affects its growth and reproduction of polypides in mice, which indicates the important role of cathepsin D in *S. mansoni* development [55]. Through proteomic analysis of the acidocalcisomes of *Trypanosoma brucei*, researchers screened acid phosphatase and the functional analysis of this enzyme through RNAi showed that it was involved in growth and development [56]. These results indicated that cathepsin B, cathepsin D and acid phosphatase are involved in embryonic development and play essential roles in growth and reproduction.

The dynamic changes and functions of these three genes in embryonic development have been studied, but the enzyme-enzyme and enzyme-vitellin interactions are still not clear. The interaction of cathepsin B and acid phosphatase has been found in *P. americana* and *M. domestica* and the hydrolysis of vitellin is increased when both enzymes are present [20, 23]. In *R. prolixus*, acid phosphatase can hydrolyze polyphosphate (polyP) to abolish the inhibitory effect of polyP on cathepsin D, which could facilitate the degradation of vitellin by cathepsin D [18, 19]. The same interaction of the two enzymes is found in *D. maxima* [25]. In future work, we will investigate the relationships between the three enzymes and identify their effects on vitellin and embryogenesis to reveal the reasons for the differences between the two reproductive mechanisms.

## Conclusions

The dynamic changes in cathepsin B, cathepsin D and acid phosphatase during the embryonic development of parthenogenetic *H. longicornis* have been identified. The three enzymes all play an important role in embryonic development, but the expression patterns and changes in the activity of the enzymes in the bisexual and parthenogenetic populations are different. After the knockdown of the genes encoding the enzymes, oviposition was not affected, whereas the hatching rate of eggs was significantly decreased. These above results will help a better understanding of the similarities and differences in embryonic development between the bisexual and parthenogenetic populations and will contribute to the future exploration of the development of the parthenogenetic population of *H. longicornis*.

## Data Availability

The data supporting the conclusions of this article are included within the article. Raw data used or analyzed during the present study are available from the corresponding author upon reasonable request.

## Abbreviations

RH: relative humidity
L:D: light:dark photocycle
PCR: polymerase chain reaction
qPCR: quantitative real-time PCR
BCA: bicinchoninic acid
AFC: amino-4-trifluoromethyl coumarin
pNPP: p-nitrophenyl phosphate
MCA: 4-methylcoumarin-7-amide
T7: TAA TAC GAC TCA CTA TAG G
dsRNA: double-stranded RNA
RNAi: RNA interference
polyp: polyphosphate
SE: standard error
